# A Comprehensive Study of Extramural Venous Invasion in Colorectal Cancer

**DOI:** 10.1371/journal.pone.0144987

**Published:** 2015-12-15

**Authors:** David McClelland, Graeme I Murray

**Affiliations:** 1 Department of Pathology, Aberdeen Royal Infirmary, NHS Grampian, Aberdeen, United Kingdom; 2 Pathology, Division of Applied Medicine, School of Medicine, Medical Sciences and Nutrition, University of Aberdeen, Aberdeen, United Kingdom; Queen Mary Hospital, HONG KONG

## Abstract

Colorectal cancer is a common malignancy and a leading cause of cancer related death. Cancer staging following resection is key to determining any adjuvant therapy in those patients with high risk disease. In colorectal cancer, tumour stage and lymph node stage are the main pathological factors which have been considered to influence outcome. Increasing emphasis is now being placed on other factors, especially the presence of extramural venous invasion (EMVI). It is important to understand the relationship of EMVI with other pathological factors and to confirm that in an individual centre that EMVI is being detected at an appropriate rate and is of prognostic significance. This comprehensive study assesses the reporting and prognostic significance of EMVI in a single centre, using prospectively collected data from histopathology reports of a cohort of 2405 patients who underwent surgery for colorectal cancer over a nine year period. Overall, EMVI was reported in 27.9% of colorectal cancer excision specimens. In tumours (n = 1928) that had not received neoadjuvant therapy, the presence of EMVI varied significantly depending on tumour site (χ^2^ = 12.03, p<0.005), tumour stage (χ^2^ = 268.188, p<0.001), lymph node stage (χ^2^ = 294.368, p<0.001) and Dukes’ stage (χ^2^ = 253.753, p<0.001). Multivariate analysis confirmed EMVI as a significant independent prognostic indicator (p<0.001). In conclusion, the presence of EMVI as an independent prognostic indicator is shown and is related to other pathological and prognostic factors. This study emphasises the requirement for the accurate identification of EMVI in colorectal cancer excision specimens and also understanding the relationship of EMVI with other prognostic factors.

## Introduction

Colorectal carcinoma is the most common malignancy of the gastrointestinal tract and is a leading cause of cancer related deaths [1]. Prognosis is predicted by the extent of tumour spread locally, the presence of lymphatic system involvement and/or metastasis to other organs which are encompassed in TNM or Dukes’ staging. Outwith these classical staging criteria, the presence of tumour cells within veins outside the bowel wall (extramural venous invasion) is an important predictor of tumour recurrence or metastasis. Consequently extramural venous invasion (EMVI) is an independent indicator of poor prognosis in colorectal carcinoma [2–11]. It should be acknowledged that in addition to that of extramural venous channels, there is an emerging role for intramural venous channels located in the submucosa or muscularis propria (intramural venous invasion, MVI) [5, 7]. However, the prognostic significance of MVI alone is still unclear [4, 12].

Whilst not directly affecting overall tumour stage, the presence of EMVI does confer high risk disease status [13, 14]. Indeed, about 25–30% of patients with lymph node negative disease die from recurrent or metastatic disease, emphasising the role of vascular spread in colorectal cancer dissemination [15–17].

Therefore, the United Kingdom Royal College of Pathologists (RCPath) has proposed a minimum rate of detection of EMVI in colorectal cancer excision specimens. This study reports on data collected from 2005 to 2013, during which time the RCPath dataset for colorectal cancer (2^nd^ edition) published in 2007 recommended a frequency of detection of EMVI of 20% [18].

EMVI was defined by Talbot and colleagues as the presence of tumour within an endothelium-lined space that is either surrounded by a rim of muscle or contains red blood cells [19]. Additionally, the so called orphan artery sign is suggestive; where a rounded or elongated tumour profile that is not in direct continuity with the advancing tumour margin is identified adjacent to an artery, especially when no accompanying vein can be seen [3]. Whilst it is possible to detect EMVI on a haematoxylin and eosin stained slide, the application of ancillary techniques such as elastin stains or immunohistochemistry have been shown to significantly increase detection rates [2, 7, 8, 16, 20–24].

This comprehensive study assesses the trend in the evaluation of EMVI in a large cohort of patients with colorectal cancer over a nine year period in a single centre, focusing on its prognostic significance with respect to other colorectal cancer staging criteria and potential confounding factors.

## Materials and Methods

### Study population

This study included 2405 patients who had their colorectal cancer pathology resection specimens reported by the Department of Pathology, Aberdeen Royal Infirmary from 2005–2013. The Department of Pathology at Aberdeen Royal Infirmary is a regional pathology centre receiving pathology specimens from four acute hospitals over three health authorities. Aberdeen Royal Infirmary (an academic teaching hospital centre) and Dr Gray’s Hospital, Elgin (a district general hospital) are both in NHS Grampian, while Balfour Hospital, Kirkwall (NHS Orkney) and Gilbert Bain Hospital, Lerwick (NHS Shetland) are both remote and rural hospitals. Approximately 75% of the colorectal cancer surgery is performed at Aberdeen Royal Infirmary. Relevant pathological data was extracted at the time of the weekly colorectal cancer multidisciplinary team meeting from the pathology reports of the resected colorectal cancer excision specimens and a database constructed.

The database was compiled using prospectively collected histopathological data from colorectal cancers resected between 2005 and 2013. The information recorded in this database includes age, gender, year of operation, administration of neoadjuvant therapy, whether the tumour was screen detected, tumour site, tumour differentiation, tumour (T) stage, the presence of EMVI, total number of lymph nodes examined, number of lymph nodes involved by metastatic tumour, lymph node (N) stage and Dukes’ stage. Information for each parameter was available for every patient. Additionally, survival data (all-cause mortality) was available for a subset of 1004 patients (number of deaths = 243). The histopathology of all the cases in this database were reported according to the criteria set out in the RCPath dataset for the reporting of colorectal cancer excision specimens (2^nd^ edition 2007–14) which incorporates guidance from TNM5 and all cases were also subject to multi-disciplinary review [18]. Throughout this period of time NHS Grampian has been a centre for the NHS Scotland bowel screening programme (2000–2006, pilot centre for evaluation of programme, 2007-part of national programme following its implementation throughout Scotland).

### Assessment of EMVI

**Fig 1:**
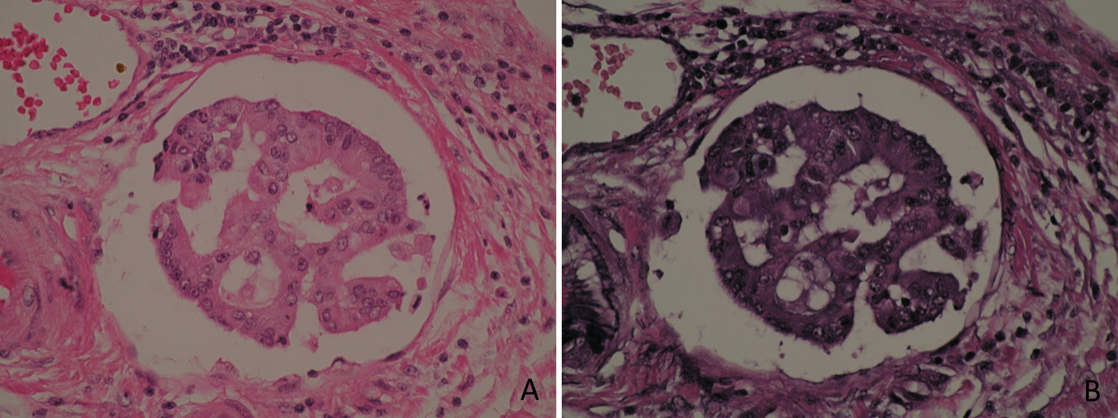
EMVI in colorectal cancer. (A) Haematoxylin and eosin. (B) Elastic haematoxylin and eosin.

The presence of tumour within venous structures beyond the bowel wall was assessed on haematoxylin and eosin stained sections of tumour, with the additional use of elastic haematoxylin and eosin staining in cases where EMVI was suspected but not clearly identified on microscopic examination of the haematoxylin and eosin stained sections (Fig 1).

### Statistics

The database was compiled in Excel 2007 and then imported into IBM SPSS version 21 for Windows 7^TM^ (IBM, Portsmouth, UK) to perform data analysis. Statistical comparisons between groups were performed using chi-square (χ^2^) test. Survival curves were prepared using the Kaplan-Meier method with log-rank (Mantel-Cox) analysis. Multivariate analysis with Cox regression (proportional hazard analysis) was also performed.

### Ethics

The project was carried out with ethics approval (ref. no. 08/S0801/81) from the North of Scotland research ethics committee. The research ethics committee did not require written participant consent and data was anonymised prior to analysis.

## Results

The clinico-pathological parameters collected for the dataset are summarised in Table 1.

**Table 1:** Clinico-pathological characteristics of the patients and their tumours.

		All cases (n = 2405)	No neodjuvant therapy (n = 1928)	Neoadjuvant therapy (n = 477)	Non-screen detected (n = 2077)	Bowel cancer screening detected (n = 328)
Gender	Male	54.7% (1315)	52.6% (1014)	63.1% (301)	53.6% (1113)	61.6% (202)
	Female	45.2% (1088)	47.3% (912)	36.9% (176)	46.3 (962)	38.4% (126)
	Unknown	0.1% (2)	0.1% (2)	-	0.1% (2)	-
Age	<71	51.1% (1229)	47.8% (920)	64.8% (309)	47.4% (985)	74.4% (244)
	≥71	48.8% (1173)	52% (1005)	35.2% (168)	52.5% (1089)	25.6% (84)
	Unknown	0.1% (3)	0.2% (3)	-	0.1% (3)	-
Tumour site	Proximal	41.5% (998)	51.2% (986)	2.5% (12)	42.5% (882)	35.4% (116)
	Distal	31.1% (747)	37.9% (731)	3.4% (16)	30.0% (624)	37.5% (123)
	Rectum	27.4% (660)	10.9% (211)	94.1% (449)	27.5% (571)	27.1% (89)
Tumour differentiation	Well/moderate	84.2% (2027)	86.6% (1669)	75.0% (358)	83.1% (1725)	90.9% (298)
	Poor	11.6% (278)	13.4% (259)	4.0% (19)	12.6% (262)	4.9% (16)
	n/a^1^	4.2% (100)	-	21.0% (100)	4.1% (86)	4.3% (14)
Tumour stage	T1	4.4% (106)	5.5% (106)	-	3.6% (75)	9.5% (31)
	T2	8.1% (194)	10.1% (194)	-	7.1% (147)	14.3% (47)
	T3	47.9% (1151)	59.7% (1151)	-	47.8% (993)	48.2% (158)
	T4	19.8% (477)	24.7% (477)	-	21.5% (447)	9.2% (30)
	yT0	4.5% (109)	-	22.9% (109)	4.5% (94)	4.6% (15)
	yT1	1.9% (46)	-	9.6% (46)	1.9% (39)	2.1% (7)
	yT2	4.2% (101)	-	21.2% (101)	4.0% (84)	5.2% (17)
	yT3	8.3% (199)	-	41.7% (199)	8.5% (177)	6.7% (22)
	yT4	0.9% (22)	-	4.6% (22)	1% (21)	0.3% (1)
Lymph node stage	N0	44.0% (1059)	54.9% (1059)	-	43.0% (894)	50.3% (165)
	N1	22.3% (537)	27.9% (537)	-	22.6% (470)	20.4% (67)
	N2	13.8% (332)	17.2% (332)	-	14.4% (298)	10.4% (34)
	yN0	15.0% (361)	-	75.7% (361)	15.3% (317)	13.4% (44)
	yN1	3.4% (82)	-	17.2% (82)	3.2% (66)	4.88% (16)
	yN2	1.4% (34)	-	7.1% (34)	1.5% (32)	0.6% (2)
Dukes stage	A	15.4% (370)	12.7% (244)	26.4% (126)	13.9% (288)	25% (82)
	B	39.5% (949)	42.3% (815)	28.1% (134)	40.3% (857)	34.1% (112)
	C	41.0% (985)	45.1% (869)	24.3% (116)	41.6% (865)	36.6% (120)
	n/a^1^	4.2% (101)	-	21.2% (101)	4.2% (87)	4.3% (14)
EMVI	Yes	27.9% (670)	31.4% (606)	13.4% (64)	28.6% (593)	23.5% (77)
	No	72.1% (1735)	68.6% (1322)	86.6 (413)	71.4% (1484)	76.52% (251)

^1^ n/a no residual tumour in the excision specimen and indicates a complete pathological response (i.e. ypT0N0) to neoadjuvant therapy

In this study, EMVI was reported in 27.9% of all colorectal cancer cases (n = 2405). EMVI was reported in 31.4% of cases where no neoadjuvant therapy had been received (n = 1928) compared with 13.4% in cases receiving neoadjuvant therapy (n = 477). Cases were subsequently grouped for analysis into those that had received neoadjuvant therapy or those that had not and cases that were screen detected or were not. Further subdivision into stage (primary tumour stage, lymph node stage or Dukes’ stage) and site of primary tumour was carried out for analysis.

### The relationship of EMVI with tumour site

Tumours were grouped into the categories of proximal (appendix, caecum, ascending colon, hepatic flexure and transverse colon tumours), distal (splenic flexure, descending colon and sigmoid colon tumours) and rectum depending on their anatomical location.

**Fig 2:**
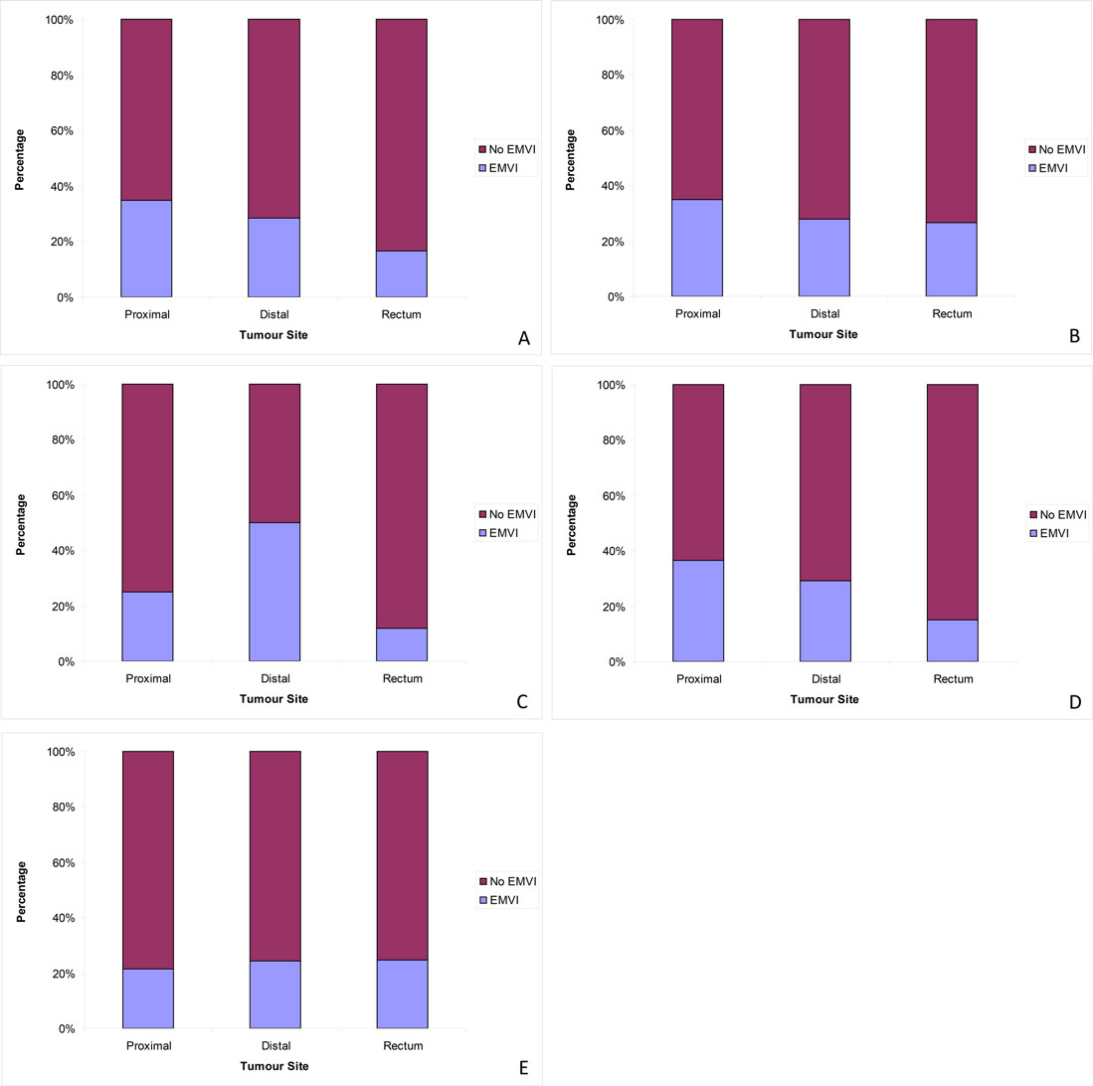
Frequency of EMVI in relation to site of primary tumour. (A) All cases. (B) Cases that had not received neoadjuvant therapy. (C) Cases that had received neoadjuvant therapy. (D) Non-screen-detected cases. (E) Bowel cancer screening detected cases.

When all cases of colorectal cancer were considered (n = 2405), the frequency of EMVI differed significantly depending on the primary tumour site. EMVI was reported in 35.0% of proximal, 29.0% of distal and 17.0% of rectum cases (χ^2^ = 66.83, p<0.001) (Fig 2).

In cases that had not received neoadjuvant therapy (n = 1928), the frequency of EMVI differed significantly depending on site of primary tumour. EMVI was reported in 35.0% of proximal, 28.0% of distal and 26.5% of rectum cases (χ^2^ = 12.03, p<0.005). For cases that had received neoadjuvant therapy (n = 477), the frequency of EMVI differed significantly depending on site of primary tumour. EMVI was reported in 25.0% of proximal, 50.0% of distal and 11.8% of rectum cases (χ^2^ = 20.82, p<0.001).

When cases that had not been screen detected were analysed (n = 2077) the frequency of EMVI differed significantly depending on site of primary tumour. EMVI was reported in 36.6% of proximal, 29.3% of distal and 15.2% of rectum cases (χ^2^ = 77.97, p<0.001). Whereas, in screen detected cases (n = 328) EMVI reporting did not differ significantly between sites of primary tumour. EMVI was reported in 21.6% of proximal, 24.4% of distal and 24.7% of rectum cases (χ^2^ = 0.373, p = 0.830).

### The relationship of EMVI with tumour stage

**Fig 3:**
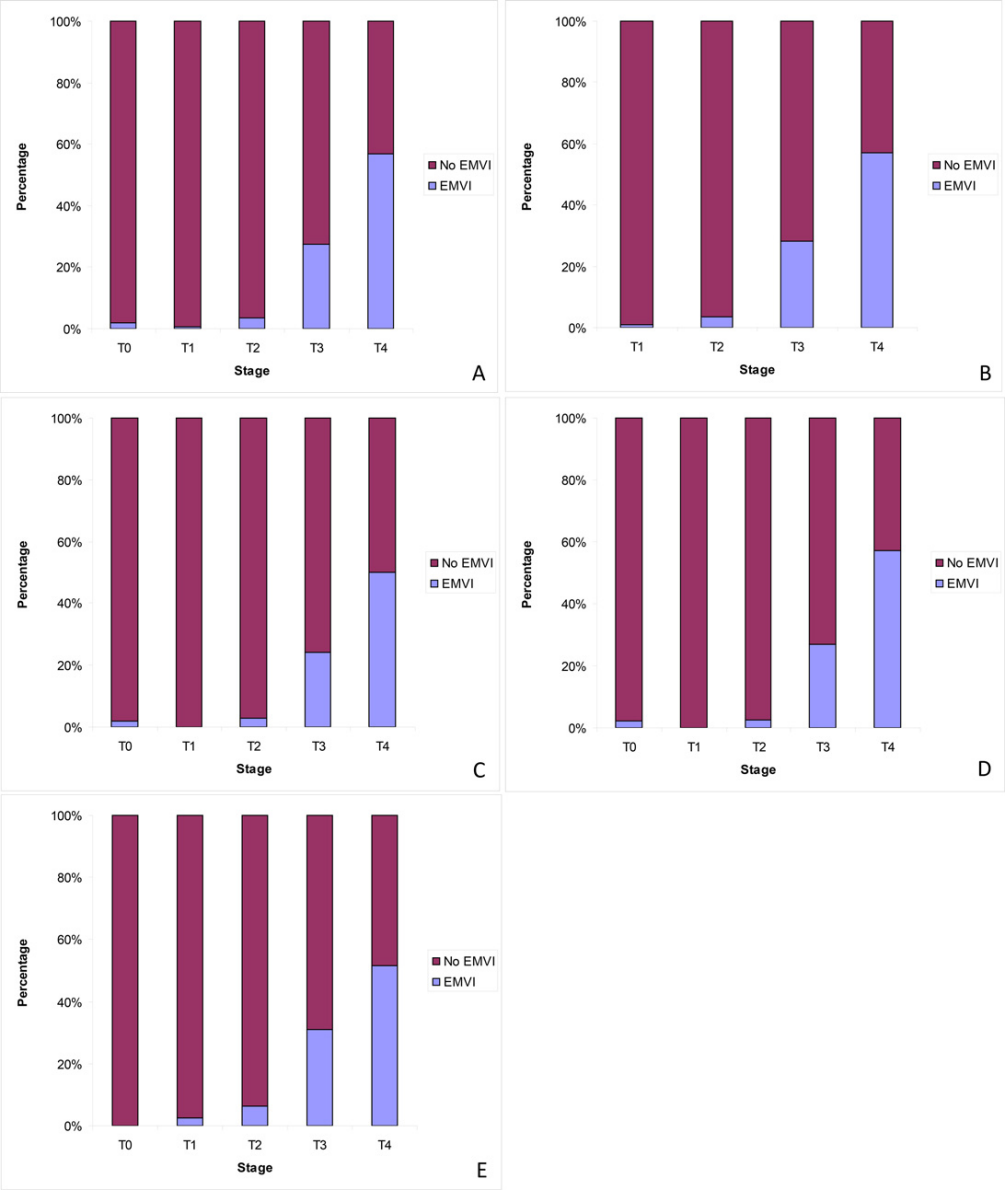
Frequency of EMVI reporting per tumour (T) stage of primary tumour. (A) All cases. (B) Cases that had not received neoadjuvant therapy. (C) Cases that had received neoadjuvant therapy. (D) Non-screen-detected cases. (E) Bowel cancer screening detected cases. T0 represents cases in which there was a complete pathological response of the primary tumour to neoadjuvant therapy.

The rate of EMVI also varied significantly depending on tumour (T) stage of primary tumour when all cases were considered. EMVI was reported in 1.8% of T0 (complete pathological response of primary tumour to neoadjuvant therapy), 0.7% of T1, 3.4% of T2, 27.6% of T3 and 56.9% of T4 tumours (χ^2^ = 390.212, p<0.001) (Fig 3).

In cases that had not received neoadjuvant therapy, EMVI was present in 0.9% of T1 tumours, 3.6% of T2 tumours, 28.2% of T3 tumours and 57.2% T4 tumours (χ^2^ = 268.188, p<0.001). For cases that had received neoadjuvant therapy, the presence of EMVI differed significantly depending on T stage of primary tumour. EMVI was reported in 1.8% of T0, 0% of T1, 3.0% of T2, 24.1% of T3 and 50% of T4 cases (χ^2^ = 74.173, p<0.001).

When cases that had not been screen detected were analysed, the presence of EMVI differed significantly depending on T stage of primary tumour. EMVI was reported in 2.1% of T0, 0% of T1, 2.6% of T2, 27.1% of T3 and 57.3% of T4 cases (χ^2^ = 344.376, p<0.001). There was also a significant difference in the frequency of EMVI depending on T stage of primary tumour in screen detected cases. EMVI was reported in 0% of T0, 2.6% of T1, 6.2% of T2, 31.1% of T3 and 51.6% of T4 cases (χ^2^ = 43.866, p<0.001).

### The relationship of EMVI with lymph node stage

**Fig 4:**
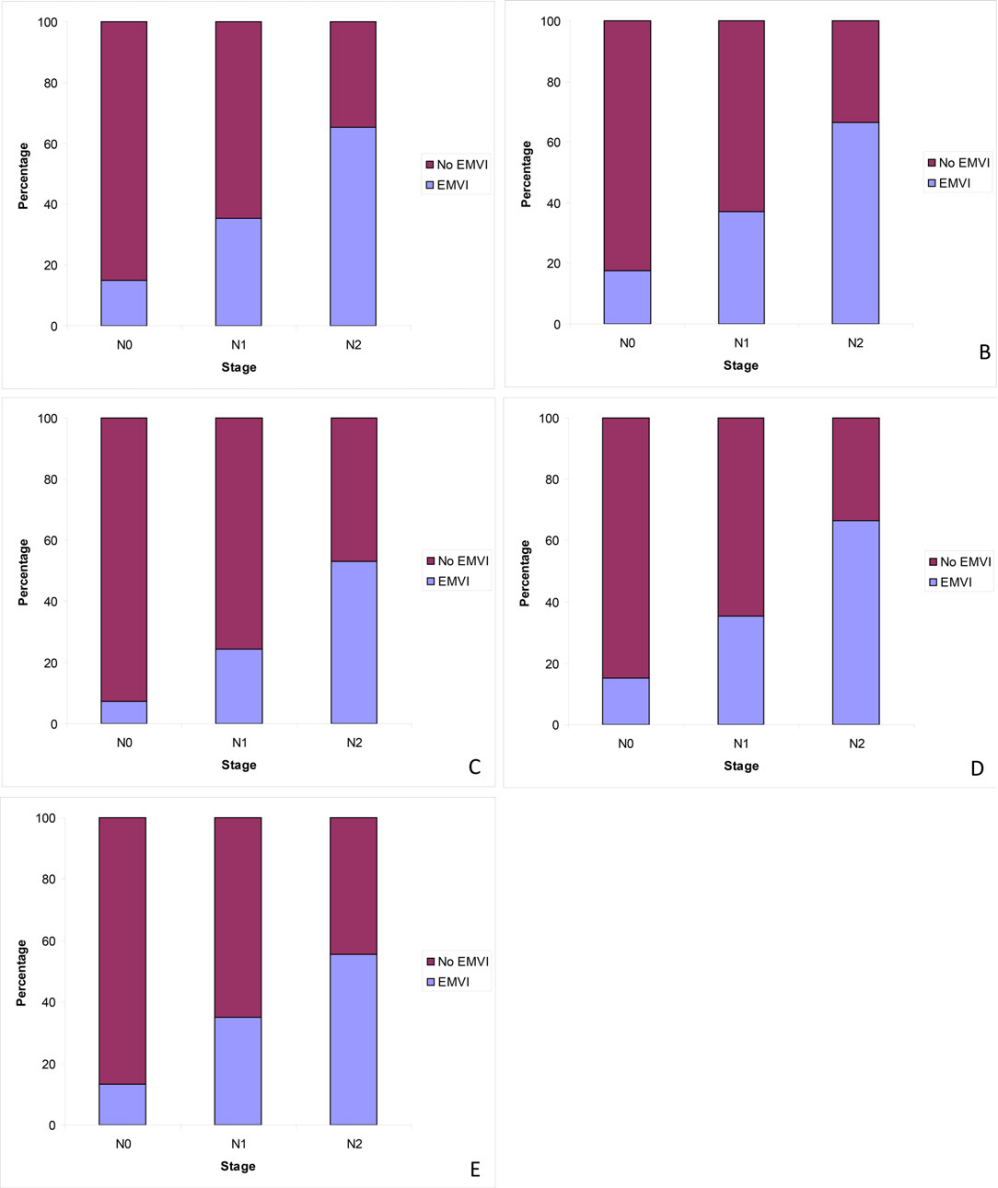
Frequency of EMVI reporting per lymph node (N) stage of primary tumour. (A) All cases. (B) Cases that had not received neoadjuvant therapy. (C) Cases that had received neoadjuvant therapy. (D) Non-screen-detected cases. (E) Bowel cancer screening detected cases.

When all cases were considered, the presence of EMVI differed significantly depending on lymph node (N) stage of primary tumour. EMVI was reported in 14.9% of N0, 35.5% of N1 and 65.3% of N2, cases (χ^2^ = 392.878, p<0.001) (Fig 4).

In cases that had not received neoadjuvant therapy, there was a significant difference in EMVI rate depending on N stage, with EMVI present in 17.5% of N0, 37.2% of N1 and 66.6% of N2 tumours respectively (χ^2^ = 294.368, p<0.001). When cases that had received neoadjuvant therapy are considered, there is also a significant difference in EMVI rate depending on N stage. EMVI was present in 7.2% of N0, 24.4% of N1 and 52.9% of N2 tumours respectively (χ^2^ = 66.222, p<0.001).

Cases that were not screen detected showed a significant difference in EMVI rate depending on N stage, with EMVI present in 15.1% of N0, 35.6% of N1 and 66.4% of N2 tumours respectively (χ^2^ = 351.707, p<0.001). A significant difference in the frequency of EMVI depending on N stage was also seen in cases that had been screen detected, with EMVI present in 13.4% of N0, 34.9% of N1 and 55.6% of N2 cases respectively (χ^2^ = 38.513, p<0.001).

### The relationship of EMVI with Dukes’ stage

**Fig 5:**
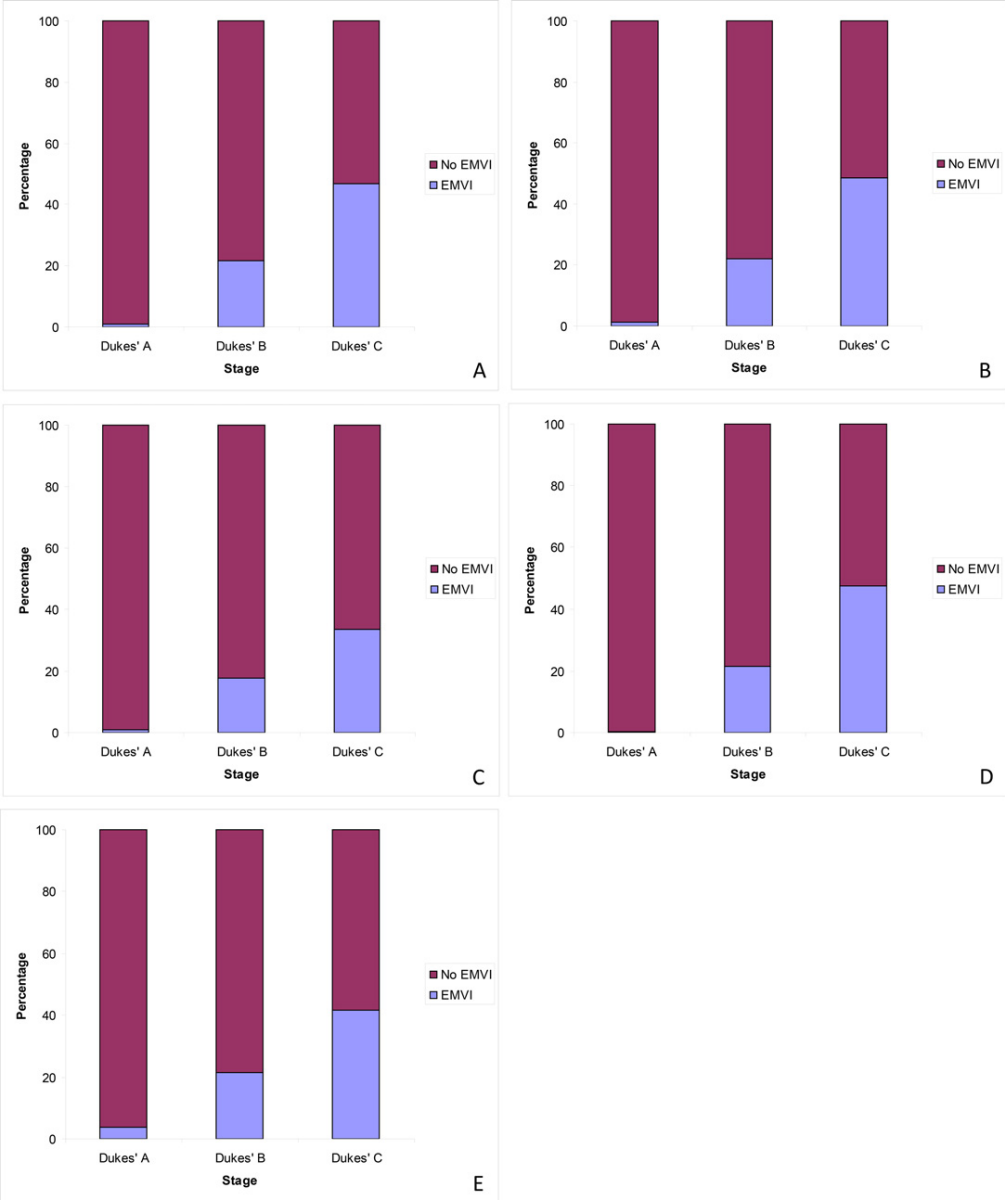
Frequency of EMVI reporting per Dukes’ stage of primary tumour. (A) All cases. (B) Cases that had not received neoadjuvant therapy. (C) Cases that had received neoadjuvant therapy. (D) Non-screen-detected cases. (E) Bowel cancer screening detected cases.

The frequency of EMVI reporting in different Dukes’ stage tumours was also considered. When all cases were analysed, EMVI was reported in 1.1% of Dukes’ A cases, 21.6% of Dukes’ B cases and 46.8% of Dukes’ C cases. (χ^2^ = 365.373, p<0.001) (Fig 5).

For cases that had not received neoadjuvant therapy EMVI was reported in 1.2% of Dukes’ A cases, 22.2% of Dukes’ B cases and 48.6% of Dukes’ C cases (χ^2^ = 253.753, p<0.001). In cases that had received neoadjuvant therapy, EMVI was reported in 0.8% of Dukes’ A cases, 17.9% of Dukes’ B cases and 33.6% of Dukes’ C cases (χ^2^ = 76.022, p<0.001).

In non-screen detected cases, EMVI was reported in 0.3% of Dukes’ A cases, 21.6% of Dukes’ B cases and 47.5% of Dukes’ C cases (χ^2^ = 319.239, p<0.001). For those cases that were screen detected, EMVI was reported in 3.7% of Dukes’ A, 21.4% of Dukes’ B and 41.7% of Dukes’ C tumours (χ^2^ = 44.586, p<0.001).

### EMVI and survival analysis

**Fig 6:**
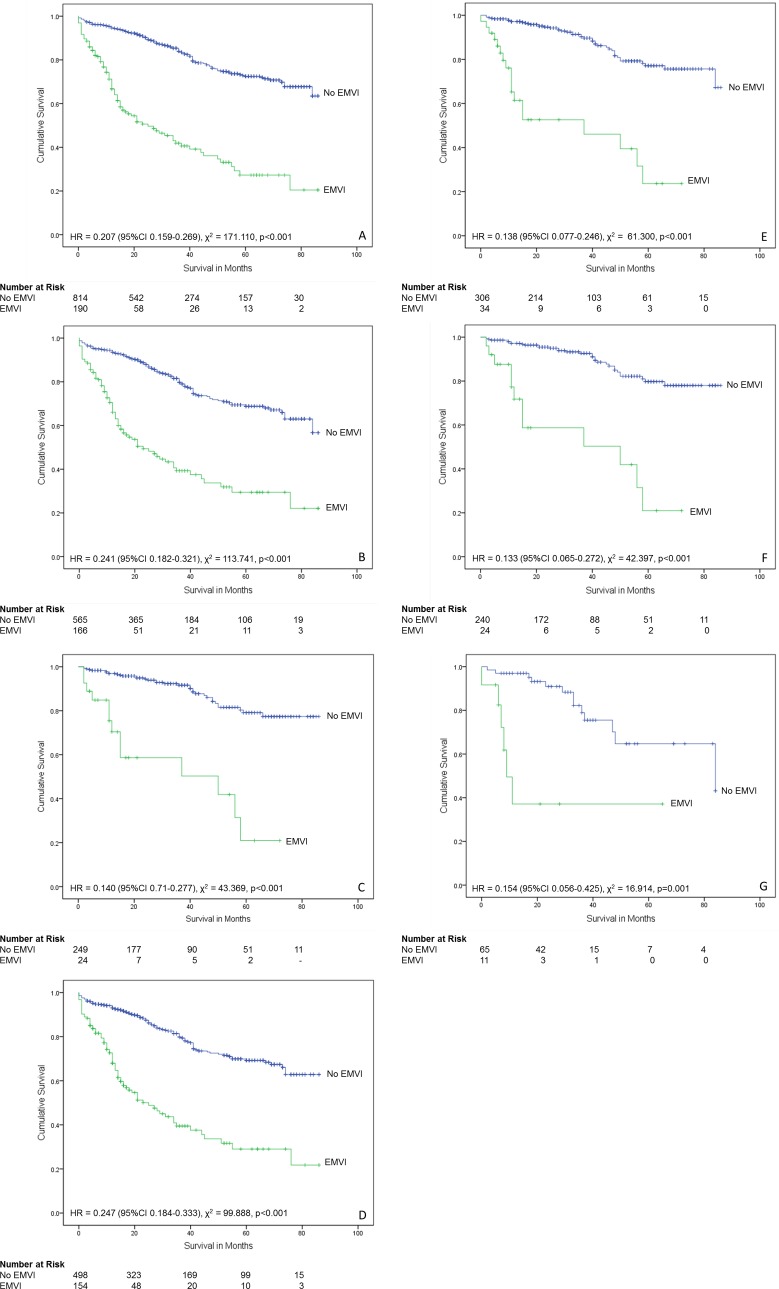
Relationship between extramural venous invasion and survival. (A) All patients (n = 1004). (B) Patients who did not receive neoadjuvant therapy (n = 731). (C) Patients who received neoadjuvant therapy (n = 273). (D) Colon cancer cases that did not receive neoadjuvant therapy (n = 653). (E) All rectal cancer cases (n = 396). (F) Rectal cancer cases that received neoadjuvant therapy (n = 318). (G) Rectal cancer cases that did not receive neoadjuvant therapy (n = 78).

Follow-up data were available for all-cause mortality over an 86 month period in 1004 of the 2405 patients in the database (41.7%). Of these, 190 (18.9%) cases had EMVI and 814 (81.1%) did not. When all cases were considered, EMVI reduced patient survival over time and these survival distributions were statistically different (χ^2^ = 117.110, p<0.001, HR = 0.207 (95% CI 0.159–0.269), Mantel-Cox log-rank test) (Fig 6).

Survival data was available for 731 of the cases that had not received neoadjuvant therapy. Of these, 166 (22.7%) had EMVI and 565 (77.3%) did not. The presence of EMVI was associated with significantly reduced patient survival over time (χ^2^ = 113.741, p<0.001, HR = 0.241 (95% CI 0.182–0.321, Mantel-Cox log-rank test).

Survival data was available for 273 of the cases that had received neoadjuvant therapy. Of these, 24 (8.8%) had EMVI and 249 (91.2%) did not. The presence of EMVI was associated with significantly reduced patient survival over time (χ^2^ = 43.639, p<0.001, HR = 0.140 (95% CI 0.071–0.277, Mantel-Cox log-rank test).

When the cases of colon cancer that had not received neoadjuvant therapy (n = 653) were considered, 154 (23.6%) had EMVI and 499 (76.4%) did not. The presence of EMVI was associated with significantly reduced patient survival over time (χ^2^ = 99.888, p<0.001, HR = 0.247 (95% CI 0.184–0.333, Mantel-Cox log-rank test).

When all rectal cancers (n = 306) were considered, EMVI was present in 34 (11.1%) and absent in 359 (89.9%) of cases. The presence of EMVI was associated with significantly reduced patient survival over time (χ^2^ = 61.300, p<0.001, HR = 0.138 (95% CI 0.077–0.246, Mantel-Cox log-rank test). Selection of rectal cancer cases that had received neoadjuvant therapy (n = 262) showed that 22 cases (8.4%) had EMVI and 240 cases (91.6%) did not. The presence of EMVI was associated with significantly reduced patient survival over time (χ^2^ = 42.397, p<0.001, HR = 0.133 (95% CI 0.065–0.272, Mantel-Cox log-rank test). A total of 78 rectal cancers had not received neoadjuvant therapy. Of these, 12 (15.4%) had EMVI and 66 (84.6%) did not. The presence of EMVI was associated with significantly reduced patient survival over time (χ^2^ = 16.914, p = 0.001, HR = 0.154 (95% CI 0.056–0.425, Mantel-Cox log-rank test).

**Fig 7:**
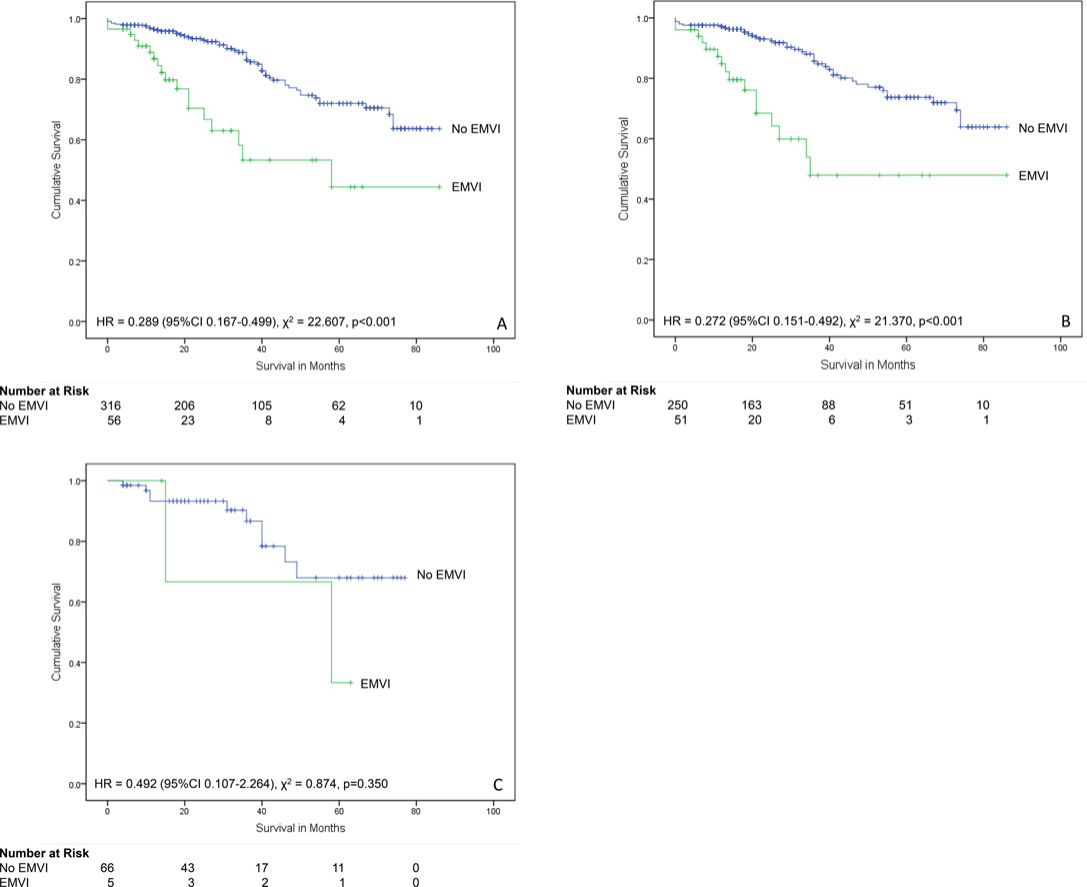
Relationship between extramural venous invasion and survival in Dukes’ B cancers. (A) All patients (n = 372). (B) Patients who did not receive neoadjuvant therapy (n = 301). (C) Patients who received neoadjuvant therapy (n = 71).

When all cases of Dukes’ B colorectal cancers were analysed (n = 372), EMVI was present in 56 (15.1%) and not present in 316 (84.9%). The presence of EMVI was associated with significantly reduced patient survival over time (χ^2^ = 22.607, p<0.001, HR = 0.289 (95% CI 0.167–0.499, Mantel-Cox log-rank test) (Fig 7).

For Dukes’ B cases that had not received neoadjuvant therapy (n = 301), 51 (16.9%) had EMVI and 250 (83.1%) did not. The presence of EMVI was associated with significantly reduced patient survival over time (χ^2^ = 21.370, p<0.001, HR = 0.272 (95% CI 0.151–0.492, Mantel-Cox log-rank test).

Survival data was available for 71 Dukes’ B colorectal cancer cases that had received neoadjuvant therapy. A total of 5 (7.0%) had EMVI and 66 (93%) did not. The presence of EMVI was not associated with significantly reduced patient survival over time (χ^2^ = 0.874, p = 0.350, HR = 0.492 (95% CI 0.107–2.264, Mantel-Cox log-rank test).

**Fig 8:**
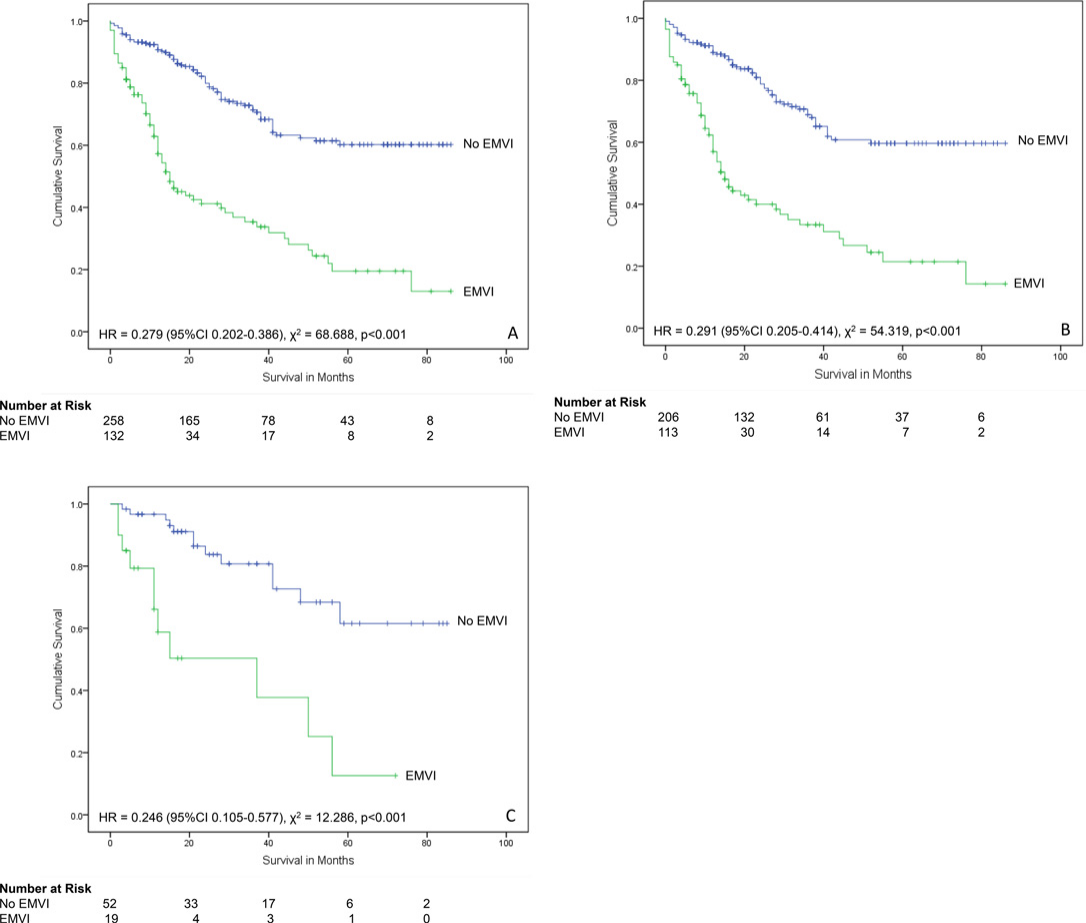
Relationship between extramural venous invasion and survival in Dukes’ C cancers. (A) All patients (n = 390). (B) Patients who did not receive neoadjuvant therapy (n = 319). (C) Patients who received neoadjuvant therapy (n = 71).

When all cases of Dukes’ C colorectal cancers were analysed (n = 390), EMVI was present in 132 (33.0%) and not present in 267 (67.0%). The presence of EMVI was associated with significantly reduced patient survival over time (χ^2^ = 68.688, p<0.001, HR = 0.279 (95% CI 0.202–0.386, Mantel-Cox log-rank test) (Fig 8).

For Dukes’ C cases that had not received neoadjuvant therapy (n = 319), 113 (35.4%) had EMVI and 206 (64.6%) did not. The presence of EMVI was associated with significantly reduced patient survival over time (χ^2^ = 54.319, p<0.001, HR = 0.291 (95% CI 0.205–0.414, Mantel-Cox log-rank test).

Survival data was available in 71 Dukes’ C colorectal cancer cases that had received neoadjuvant therapy. A total of 19 (26.8%) had EMVI and 52 (73.2%) did not. The presence of EMVI was associated with significantly reduced patient survival over time (χ^2^ = 12.286, p<0.001, HR = 0.246 (95% CI 0.105–0.577, Mantel-Cox log-rank test).

**Fig 9:**
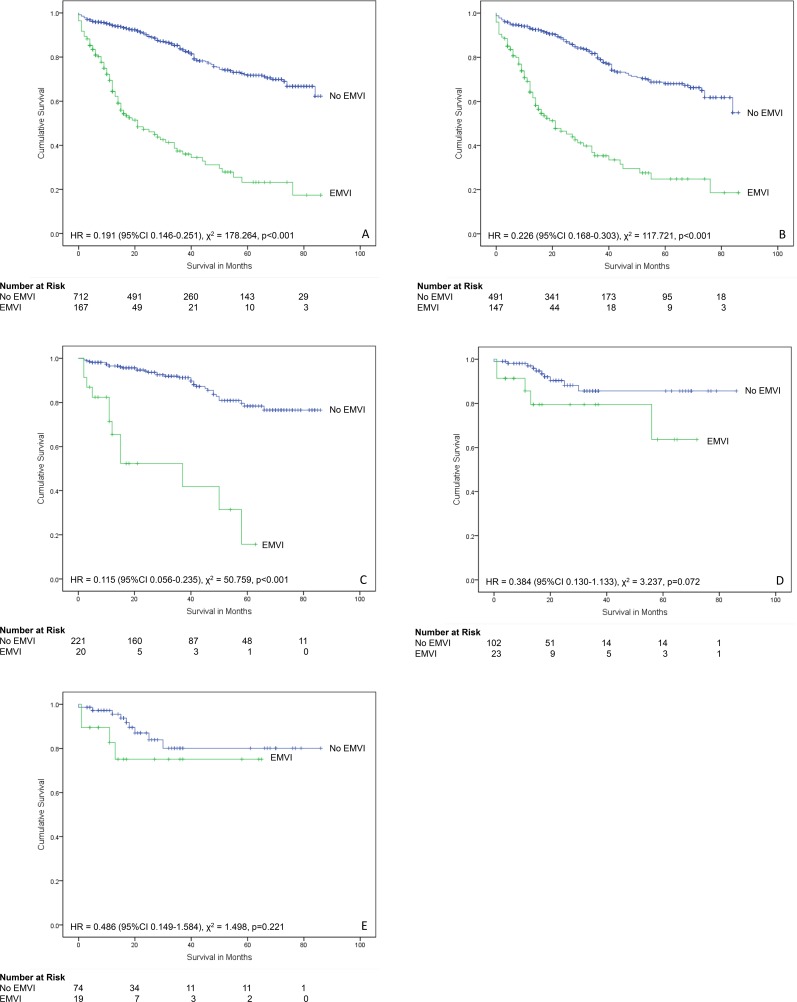
Relationship between extramural venous invasion and survival and screening in colorectal cancers. (A) All non-screen detected cancer patients (n = 879). (B) Non-screen-detected cancer patients who did not receive neoadjuvant therapy (n = 638). (C) Non-screen-detected cancer patients who received neoadjuvant therapy (n = 241). (D) All bowel cancer screening detected cases (n = 125). (E) Bowel cancer screening detected patients who did not receive neoadjuvant therapy (n = 93).

There were 879 colorectal cancer cases in the non-screen-detected group for which survival data was available. Of these, 167 (19.0%) had EMVI and 712 (81.0%) did not. The presence of EMVI was associated with significantly reduced patient survival over time (χ^2^ = 178.264, p<0.001, HR = 0.191 (95% CI 0.146–0.251, Mantel-Cox log-rank test). There were 638 non-screen-detected colorectal cancers that had not received neoadjuvant therapy. Of these, 147 (23.0%) had EMVI and 491 (77%) did not. The presence of EMVI was associated with significantly reduced patient survival over time (χ^2^ = 117.721, p<0.001, HR = 0.226 (95% CI 0.168–0.303, Mantel-Cox log-rank test). A total of 241 non-screen-detected colorectal cancer cases that had received neoadjuvant therapy were available for survival analysis. Twenty cases (8.3%) had EMVI and 221 cases (91.7%) did not. The presence of EMVI was associated with significantly reduced patient survival over time (χ^2^ = 50.759, p<0.001, HR = 0.115 (95% CI 0.056–0.235, Mantel-Cox log-rank test) (Fig 9).

Of the bowel cancer screening detected cases in which survival data was available (n = 125), 23 (18.4%) had EMVI and 102 (81.6%) did not. In this group, EMVI was not associated with a significantly reduced patient survival over time (χ^2^ = 3.237, p = 0.072, HR = 0.384 (95% CI 0.130–1.133, Mantel-Cox log-rank test). Within this group, 93 had not received neoadjuvant therapy. 19 (20.4%) had EMVI and 74 (79.6%) did not. There was no significant reduction in patient survival over time in this group (χ^2^ = 1.498, p = 0.221, HR = 0.486 (95% CI 0.149–1.584, Mantel-Cox log-rank test).

### Multivariate analysis

Multi-variate analysis showed that EMVI was a highly significant independent prognostic factor (p<0.001) when other tumour dependent factors including tumour stage, lymph node stage, Dukes stage, tumour site and degree of tumour differentiation as well as patient specific factors of age and gender are considered (Tables 2 and 3).

**Table 2:** The relationship between clinico-pathological characteristics and overall survival, when tumour stage and lymph node status are covariates and primary tumour site is grouped as proximal, distal or rectum.

	All cases				Cases that had not received neoadjuvant therapy				Cases that had received neoadjuvant therapy			
	Wald value	p-value	Hazard ration	95% CI	Wald value	p-value	Hazard ratio	95% CI	Wald value	p-value	Hazard ratio	95% CI
EMVI (present, absent)	47.415	<0.001	0.357	0.266–0.478	39.174	<0.001	0.364	0.265–0.499	11.154	0.001	0.256	0.115–0.570
Age (<71, ≥71)	46.731	<0.001	2.595	1.974–3.410	36.725	<0.001	2.572	1.895–3.492	9.739	0.002	2.837	1.474–5.462
Tumour stage (pT0, pT1, pT2, pT3, pT4)	46.006	<0.001	0.384	0.000–0.528	39.757	<0.001	0.390	0.156–0890	3.079	0.545	0.335	0.000–1.399
Lymph node stage (pN0, pN1, pN2)	30.536	<0.001	0.693	0.258–0.977	24.985	<0.001	0.648	0.252–0.937	5.369	0.068	1.821	0.234–5.241
Tumour site (proximal, distal, rectum)	2.669	0.263	0.843	0.500–1.201	3.823	0.148	0.729	0.356–1.213	1.896	0.387	3.147	0.260–16.32
Tumour differentiation (well/moderate, poor)	1.100	0.577	0.827	0.000–1.180	0.004	0.953	0.988	0.672–1.453	9.583	0.008	0.178	0.000–0.531
Gender (M, F)	0.000	0.985	1.002	0.775–1.926	0.272	0.602	1.078	0.813–1.428	0.087	0.768	0.900	0.445–1.820

**Table 3:** The relationship between clinico-pathological characteristics and overall survival, when Dukes’ stage is a covariate and primary tumour site is grouped as proximal, distal or rectum.

	All cases				Cases that had not received neoadjuvant therapy				Cases that had received neoadjuvant therapy			
	Wald value	p-value	Hazard ratio	95% CI	Wald value	p-value	Hazard ratio	95% CI	Wald value	p-value	Hazard ratio	95% CI
EMVI (present, absent)	73.655	<0.001	0.293	0.222–0.388	62.550	<0.001	0.296	0.219–0.400	12.239	<0.001	0.252	0.116–0.545
Age (<71, ≥71)	47.278	<0.001	2.603	1.982–3.418	36.090	<0.001	2.526	1.867–3.417	10.447	0.001	2.976	1.538–5.759
Dukes’ stage (A, B, C)	37.981	<0.001	9.910	0.318–105.506	27.331	<0.001	0.776	0.519–1.062	8.594	0.035	17.280	0.230–320.371
Tumour site (proximal, distal, rectum)	3.612	0.057	1.023	0.629–1.447	2.092	0.351	0.990	0.667–1.236	5.111	0.078	4.978	0.498–23.083
Tumour differentiation (well/moderate, poor)	3.054	0.217	0.788	0.110–5.673	0.565	0.452	0.866	0.596–1.260	10.678	0.005	0.280	0.031–2.567
Gender (M, F)	0.000	0.985	1.002	0.776–1.296	0.278	0.598	1.079	0.814–1.429	0.078	0.780	0.905	0.447–1.829

EMVI was not a significant prognostic cofactor in screen detected cases but was in non-screen detected cases (Tables 4 and 5).

**Table 4:** The relationship between clinico-pathological characteristics and overall survival when tumour and lymph node stage are covariates and primary tumour site is grouped as proximal, distal or rectum.

	Non-screen detected cases				Bowel cancer screening detected cases			
	Wald value	p-value	Hazard ratio	95% CI	Wald value	p-value	Hazard ratio	95% CI
EMVI (present, absent)	50.752	<0.001	0.327	0.241–0.445	0.177	0.674	0.723	0.160–3.268
Tumour stage (pT0, pT1, pT2, pT3, pT4)	42.463	<0.001	0.383	0.000–0.571	2.303	0.680	0.325	0.000–4.351
Age (<71, ≥71)	41.215	<0.001	2.527	1.904–3.354	0.459	0.498	1.646	0.390–6.949
Lymph node stage (pN0, pN1, pN2)	27.403	<0.001	0.696	0.256–0.997	3.971	0.137	0.671	0.231–2.899
Tumour site (proximal, distal, rectum)	4.231	0.121	0.741	0.450–1.072	2.201	0.333	3.319	0.231–19.977
Tumour differentiation (well/moderate, poor)	1.378	0.502	0.802	0.000–1.160	0.465	0.792	0.520	0.000–3.403
Gender (M, F)	0.062	0.803	0.967	0.741–1.262	0.721	0.396	1.640	0.523–5.137

**Table 5:** The relationship between clinico-pathological characteristics and overall survival when Dukes’ stage is a covariate and primary tumour site is grouped as proximal, distal or rectum.

	Non-screen detected cases				Bowel cancer screening detected cases			
	Wald value	p-value	Hazard ratio	95% CI	Wald value	p-value	Hazard ratio	95% CI
EMVI (present, absent)	78.078	<0.001	0.267	0.199–0.358	0.318	0.573	0.674	0.171–2.654
Age (<71, ≥71)	39.433	<0.001	2.467	1.861–3.270	2.543	0.111	3.109	0.771–12.537
Dukes’ stage (A, B, C)	32.522	<0.001	8.128	0.272–85.485	6.513	0.039	0.230	0.020–1.283
Tumour differentiation (well/moderate, poor)	3.411	0.182	0.617	0.086–4.422	0.277	0.871	0.622	0.000–3.638
Tumour site (proximal, distal, rectum)	0.868	0.648	0.918	0.569–1.315	1.720	0.423	3.197	0.395–18.214
Gender (M, F)	0.098	0.755	0.958	0.735–1.251	2.356	0.125	2.450	0.780–7.691

## Discussion

It is important for individual centres reporting colorectal excision specimen to demonstrate an appropriate frequency of detection of pathological prognostic factors taking account of confounding factors e.g. proportion of patients receiving neoadjuvant therapy and proportion of cases identified through a bowel cancer screening programme. Individual centres should also demonstrate that the pathological factors that are assumed to be prognostically significant are indeed prognostically significant in their population. This large study from a single centre utilises prospectively collected data to comprehensively assess the evaluation of EMVI in colorectal cancer resection specimens over a nine year period and to consider potential confounding factors including neoadjuvant therapy and the influence of bowel cancer screening. It uses a prospectively collected dataset to examine in detail the significance of the reporting of EMVI on mortality in this population. All of the data derives from a single histopathology department, thereby reducing data collection and reporting bias.

There is well documented variation between centres in the demonstration of EMVI [1, 5, 7, 12, 16, 19–27]. Despite which, EMVI is recognised as an important prognostic feature [12, 16, 22, 28–30]. As such, reporting of the presence or absence of EMVI in colorectal cancer excision specimens is recommended by professional organisations including the Royal College of Pathologists (UK) and the College of American Pathologists [3, 9]. The consequence of accurate detection of EMVI is that patients with lymph node negative colorectal cancer (stage II/Dukes’ B disease) but with EMVI and/or other adverse prognostic features (including tumour perforation, serosal involvement, incomplete tumour resection) may benefit from and should be considered for adjuvant chemotherapy [14, 31, 32]. The presence of EMVI has become prognostically more relevant following the introduction in the UK of bowel cancer screening programmes, as this has resulted in the more frequent resection of lymph node negative tumours.

This study shows that when all cases are analysed, detection of EMVI exceeds the standard set by the Royal College of Pathologists over the same time period [18]. In addition, exclusion of cases in which preoperative neoadjuvant therapy had been given and which were potentially down staged, resulted in both the 2^nd^ edition RCPath standard (20%) as well as the revised 3^rd^ edition (2014) RCPath standard (30%) being surpassed [3, 18]. Albeit the data included in this study is prior to the publication of the third edition of the RCPath colorectal cancer dataset [3].

As might be expected, EMVI is more common in higher stage (T-, N- and Dukes’ stage) tumours and this study has demonstrated a stage dependent significant difference irrespective of whether the cases were screen-detected or not or if there had been neoadjuvant therapy or not. It has also shown the frequency of the identification of EMVI varied significantly depending on site of primary tumour when all cases, non-screen-detected cases, cases that had received neoadjuvant therapy and also those cases that had not. There was no significant tumour site dependent difference in the frequency of EMVI reporting in colorectal cancer cases that were detected by the bowel screening programme. It is not clear if that represent the smaller number of bowel cancer screening detected cases or reflects a difference in the biology between symptomatic colorectal cancer and screen detected cancers.

Whilst the frequency of EMVI appears closely related to tumour stage, multivariate analysis has shown that EMVI is an independent predictor of poor prognosis, as are patient age and tumour stage. This study allowed assessment of all-cause mortality in a subgroup of patients. Overall, EMVI was associated with statistically significantly reduced survival. Only in screen-detected cases (n = 125) and in Dukes’ B cases following neoadjuvant therapy (n = 71) was EMVI not associated with a significant reduction in survival. However, this lack of significance may reflect the limited amount of survival data available in those groups.

EMVI is clearly an important parameter with regards to patient prognosis and the post-operative management of colorectal cancer. Adequate reporting is therefore paramount and may be optimised with the use of ancillary techniques such as elastic haematoxylin and eosin staining [2, 7, 8, 16, 20–23]. The data presented supports the need for accurate assessment and reporting of EMVI along with other prognostic factors such as lymph node yield, lymph node ratio and relevant biomarkers to facilitate the decision making process of colorectal cancer treatment [33–37].
